# Seroepidemiologic Survey of Potential Pathogens in Obligate and Facultative Scavenging Avian Species in California

**DOI:** 10.1371/journal.pone.0143018

**Published:** 2015-11-25

**Authors:** Mary H. Straub, Terra R. Kelly, Bruce A. Rideout, Curtis Eng, Janna Wynne, Josephine Braun, Christine K. Johnson

**Affiliations:** 1 Wildlife Health Center, School of Veterinary Medicine, University of California Davis, Davis, California, United States of America; 2 Wildlife Disease Laboratories, San Diego Zoo Institute for Conservation Research, Escondido, California, United States of America; 3 Los Angeles Zoo and Botanical Gardens, Los Angeles, California, United States of America; 4 California Science Center Foundation, Los Angeles, California, United States of America; University of Lleida, SPAIN

## Abstract

Throughout the world, populations of scavenger birds are declining rapidly with some populations already on the brink of extinction. Much of the current research into the factors contributing to these declines has focused on exposure to drug residues, lead, and other toxins. Despite increased monitoring of these declining populations, little is known about infectious diseases affecting scavenger bird species. To assess potential infectious disease risks to both obligate and facultative scavenger bird species, we performed a serosurvey for eleven potential pathogens in three species of scavenging birds in California: the California condor (*Gymnogyps californianus*), turkey vulture (*Cathartes aura*) and golden eagle (*Aquila chrysaetos*). California condors were seropositive for avian adenovirus, infectious bronchitis virus, *Mycoplasma gallisepticum*, avian paramyxovirus-2, West Nile virus (WNV) and *Toxoplasma gondii*. Golden eagles were seropositive for avian adenovirus, *Chlamydophila psittaci* and *Toxoplasma gondii*, and turkey vultures were seropositive for avian adenovirus, *Chlamydophila psittaci*, avian paramyxovirus-1, *Toxoplasma gondii* and WNV. Risk factor analyses indicated that rearing site and original release location were significantly associated with a positive serologic titer to WNV among free-flying condors. This study provides preliminary baseline data on infectious disease exposure in these populations for aiding in early disease detection and provides potentially critical information for conservation of the endangered California condor as it continues to expand its range and encounter new infectious disease threats.

## Introduction

Worldwide, scavenging bird populations are rapidly declining [1–3]. Currently over half of the world’s vulture species are facing the threat of extinction [4] with several populations having already decreased by over 95% [5, 6]. In Africa, eight species of vulture have declined by an average of 62% in recent decades [7]. Scavenging bird species provide vital ecosystem services including decreasing the spread of disease, recycling of nutrients through the environment and reducing the costs associated with carcass disposal [8, 9]. As scavenging bird populations decline throughout the world, infectious diseases, including rabies, plague and canine distemper, are expected to increase [10–12]. Recent attention has focused on pharmaceutical drug residues, poisons aimed at predators, lead from ammunition and other toxins in animal remains serving as food resources [13–19], which all contribute to mortality, in addition to other causes of death such as human persecution and utility line collision [4, 16, 20]. Infectious diseases have been a less common focus of investigations and there are relatively few accounts of infectious diseases as a direct cause of mortality in avian scavengers. West Nile virus (WNV) was determined to be the cause of death for two California condors (*Gymnogyps californianus*) in California [16] and Newcastle disease virus has been implicated in the death of a bearded vulture (*Gypaetus barbatu*) in Israel [21]. Given the ongoing declines in scavenging birds, baseline information on infectious disease exposure will be useful for monitoring population health, investigating future disease-related epidemics, and informing conservation efforts needed for species in decline.

In this study, we investigated exposure to avian pathogens in three species of birds that commonly scavenge on carrion: the California condor, which is restricted to areas in northern Mexico and the western United States [22]; the turkey vulture (*Cathartes aura*), which is widespread throughout the Americas [23]; and the golden eagle (*Aquila chrysaetos*), which is found mainly in western North America [24]. Unlike California condors and turkey vultures, which are obligate scavengers, golden eagles are facultative scavengers feeding on both carrion and live prey. The California condor is currently listed as critically endangered [25] and an intensive captive breeding program initiated in the 1980s [26, 27] has succeeded in the reintroduction of free-flying condor populations in California, Arizona and Baja California, Mexico [28]. Lead poisoning has been a primary cause of mortality in condors, and, other than WNV, infectious diseases have not been reported as an important cause of morbidity or mortality in this species [16, 29, 30]. Because WNV can be fatal in California condors, the captive and free-flying populations in California are vaccinated against this disease with a recombinant DNA vaccine [31, 32] being used prior to and during our study period. Serologic titers are measured annually in captive and free-flying condors and individuals are re-vaccinated as needed. Even with vaccination, the species is vulnerable to this disease as evidenced by the WNV-related death of a condor that had been vaccinated twice against WNV [16].

Although infectious disease has not been documented as a significant cause of mortality in California condors, it is critical to understand the range of factors influencing health status in this species because loss of only a few individuals can influence population trajectories. Furthermore, populations consisting mainly of captive-reared individuals, such as California condors, could have increased vulnerability to infectious disease if they are immunologically naïve to pathogens circulating in free-flying sympatric species. Additionally, the California condor experienced a severe population bottleneck prior to extirpation in the wild and the subsequent decline in genetic variability [33, 34] can be associated with decreased resistance to disease [35–37]. Viability of small populations are highly susceptible to epidemic mortality events, and a baseline understanding of pathogen exposure in the California condor population is critical to informing management and recovery efforts, especially as condors expand their range and encounter new risks [17].

As sympatric species commonly sharing habitat and food resources with California condors, turkey vultures and golden eagles are exposed to many of the same hazards, including lead poisoning [14] and WNV [38–40]. Given the dramatic declines in many other scavenging birds, baseline data on pathogen exposure in the turkey vulture and golden eagle will be useful in evaluating current and future threats. Additionally, these species may serve as sentinels for infectious disease risk for species of conservation concern, such as California condors.

Here, in our serosurvey, we evaluated exposure to avian pathogens that have been reported to cause disease in a variety of wild bird species, including avian adenovirus, *Chlamydophila psittaci*, infectious bronchitis virus (IBV) (Arkansas (Ark), Connecticut (Conn) and Massachusetts (Mass) strains), *Mycoplasma gallisepticum*, *Mycoplasma synoviae*, avian paramyxovirus-1 (AVPM-1, Newcastle Disease virus), avian paramyxovirus-2 (AVPM-2), avian paramyxovirus-3 (AVPM-3), avian reovirus, *Toxoplasma gondii*, and WNV [21, 41–51]. Adenoviruses have been implicated in outbreaks involving three species of falcons which resulted in severe disease and death of numerous birds [46, 52], and exposure to adenoviruses has been detected in common buzzards (*Buteo buteo*) [44]. *Chlamydophila psittaci*, which can result in fatal respiratory disease in some avian species, has been previously isolated from many species of wild birds, including raptors [50, 53]. Infectious bronchitis virus, which causes severe respiratory disease in poultry is a coronavirus and genetically similar viruses have been detected in wild birds [54, 55]. *Mycoplasma gallisepticum* and *M*. *synoviae* are economically important pathogens in poultry, causing respiratory disease (*M*. *gallisepticum* and *M*. *synoviae*) and synovitis (*M*. *synoviae*). *Mycoplasma gallisepticum* has been associated with respiratory disease in peregrine falcons (*Falco peregrinus*) [56] and other wild avian species [57]. In the late 1990’s, *M*. *gallisepticum* emerged in house finches (*Haemorhous mexicanus*) in the eastern United States and has since spread across the USA [37]. Avian paramyxovirus-1, also known as Newcastle Disease virus, is most commonly associated with disease in poultry [58], but has also been found in both clinically affected [21] and healthy raptors [59] as well as many other species of wild birds [60]. Avian paramyxovirus-2 primarily affects poultry, causing respiratory and reproductive disease in turkeys and chickens [61–63]. However, APMV-2 has also been reported in several wild bird species including mallard ducks [64], passerines [65] and raptors [66]. Avian paramyxovirus-3 also causes reproductive disease in turkeys [58], and has been reported in multiple species of wild birds [43, 67]. Reoviruses are associated with many disease processes and high mortality in various wild bird species [42, 68, 69]. *Toxoplasma gondii* is a protozoal parasite that clinically affects many avian species [41, 70] and was reported to cause fatal myocarditis of a bald eagle (*Haliaeetus leucocephalus*) [71].

The objectives of our study were two-fold: first, to obtain baseline data on the seroprevalence of common avian pathogens that could pose a threat to California condors, turkey vultures and golden eagles; and second, to understand patterns in WNV serostatus among California condors, including associations with several potential predictors such as age, location, number of previous WNV vaccines and time since last WNV vaccination.

## Material and Methods

### Ethics Statement

Capture and sampling of golden eagles and turkey vultures was covered under federal and state permits (United States Geological Survey federal bird banding permit # 20431 and California Department of Fish and Game scientific collecting permit # 000221) and approved by the University of California, Davis Institutional Animal Care and Use Committee (protocol # 07–12955). Condor captures were approved by the US Fish and Wildlife Service Permit Coordinator, the US Fish and Wildlife Service California Condor Coordinator, and the US Fish and Wildlife Service Region 8 Endangered Species Division (permits # TE-026659, # TE-108507 and # TE-157291).

### Study populations and sites

**Fig 1:**
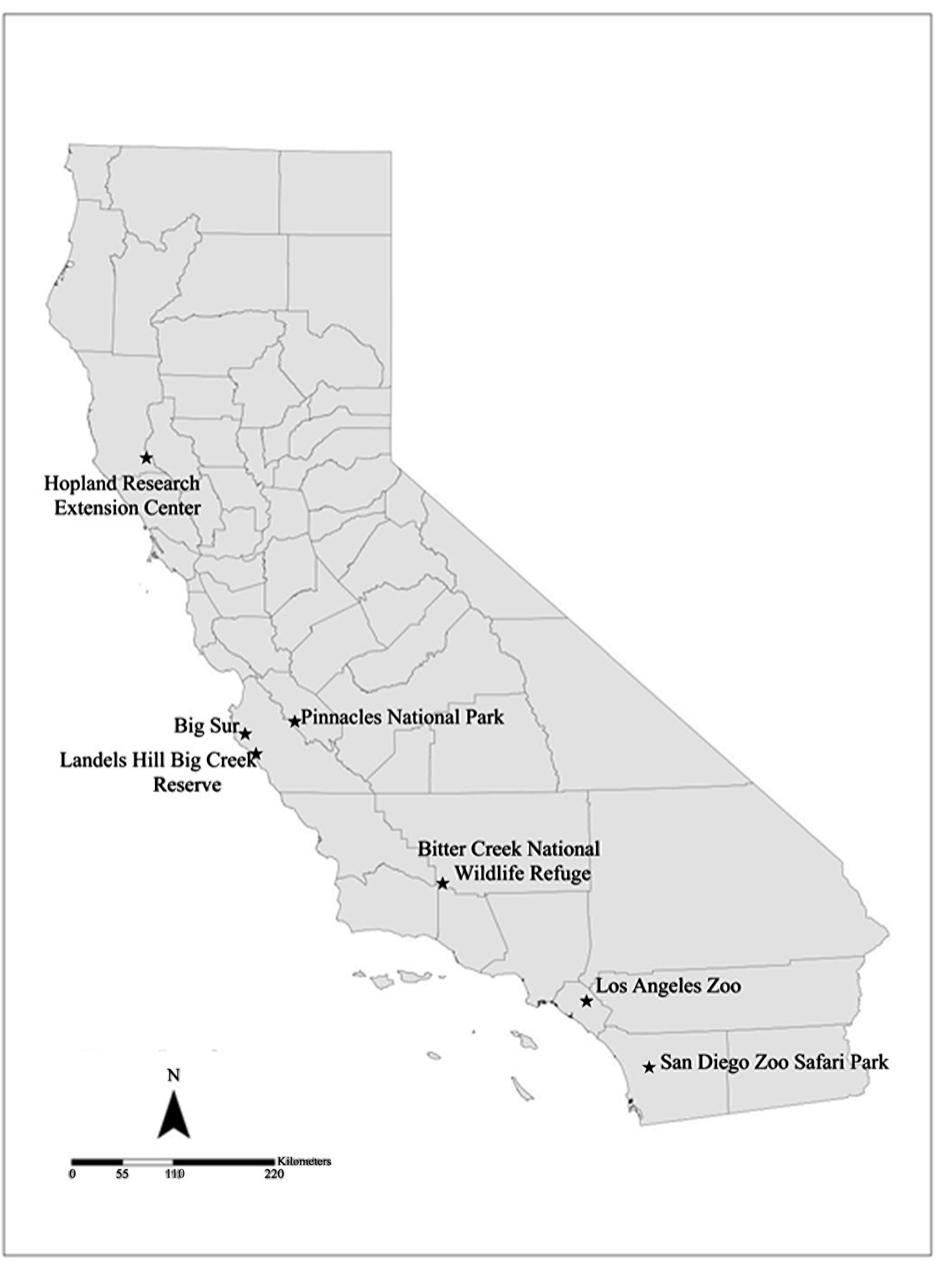
Sampling sites of California condors, golden eagles and turkey vultures in California. Sampling sites designated by black stars.

Free-flying California condors (n = 96) were sampled between September 2010 and January 2011 at three release sites in California: Big Sur in Monterey County (n = 34); Pinnacles National Park in San Benito County (n = 20); and Bitter Creek National Wildlife Refuge in Kern County (n = 42) as part of an on-going monitoring and recovery program (Fig 1). These condors were either raised in captivity and subsequently released into the wild, or raised in the wild. Because condors occasionally travel between these sites, they were classified by their original release location. Serum samples were also obtained from 28 California condors in captivity at the San Diego Zoo Safari Park (SDZSP) and 13 California condors in captivity at the Los Angeles Zoo (LAZ) during routine examinations between January 2008 and August 2012 (Fig 1). These birds were held in captivity throughout their lives and were never released to the wild prior to sample collection. All California condors were uniquely identified by patagium tags.

Golden eagle and turkey vulture blood samples were collected during capture of apparently healthy free-flying birds. Samples were obtained from 26 eagles between December 2008 and May 2009 in Kern County, California. Golden eagles were aged based on plumage [72] and categorized as either juvenile (n = 3), subadult (n = 14) or adult (n = 9). Turkey vultures were captured near Big Sur in Monterey County, and in Mendocino County, California. Samples were obtained from 34 vultures captured near Big Sur and 32 vultures captured in Mendocino County between May 2009 and July 2009. Turkey vultures were categorized into two age classes, younger than second year (Big Sur: n = 3; Mendocino: n = 2), and after second year (Big Sur: n = 29; Mendocino: n = 32), based on coloration of the head and maxilla [73]. Capture methods and animal handling procedures have been previously described [14, 74].

### Sample Collection and Diagnostic Testing

Blood was drawn from the metatarsal or brachial vein and aliquoted into additive-free, EDTA, and lithium heparin blood collection tubes (Becton Dickinson, Franklin Lakes, NJ). Samples were kept cool on ice for transport back to the University of California, Davis where they were aliquoted and stored at -80°C until shipment to the diagnostic laboratories. Serological testing for avian adenovirus, *Chlamydophila psittaci*, infectious bronchitis virus (IBV) (Arkansas (Ark), Connecticut (Conn) and Massachusetts (Mass) strains), *Mycoplasma gallisepticum*, *Mycoplasma synoviae*, avian paramyxovirus-1 (Newcastle Disease virus), avian paramyxovirus-2, avian paramyxovirus-3, and avian reovirus was performed at Texas Veterinary Medical Diagnostic Laboratory (College Station, TX) using assays optimized for poultry species. Because these assays have not been validated in condors, vultures, or eagles, we relied on cut-off titers established for poultry. Exposure to avian adenovirus was evaluated using an agar gel immunodiffusion (AGID) test. *Chlamydophila psittaci* exposure status was determined by a direct complement fixation (DCF) assay. Exposure status for the three strains of IBV was determined by hemagglutination inhibition (HI) assay. A titer of 1:16 or greater was considered positive for exposure to IBV. *Mycoplasma gallisepticum* exposure status was determined using serial tests. Samples were initially screened using a plate agglutination assay. Any samples that were positive on the first assay were then tested by HI. A titer of 1:80 or above on the HI assay was considered positive. A sample testing positive on both the plate agglutination test and HI assay was considered positive for exposure to *M*. *gallisepticum*. *Mycoplasma synoviae* exposure status was determined in the same manner as *M*. *gallisepticum*. Avian paramyxovirus 1, 2 and 3 exposure status was determined by HI. A titer of 1:16 or greater on the HI assay was considered positive for each of the three paramyxoviruses. Avian reovirus exposure status was determined using AGID. The number of individuals tested varied between pathogens due to limitations in sample volume.

Serological testing for *Toxoplasma gondii* was performed at University of California, Davis (Department of Pathology, Microbiology, and Immunology, University of California-Davis) using a *T*. *gondii* agglutination test kit (Eiken Chemical Co., LTD. Tokyo, Japan, distributed by Tanabe USA, Inc., San Diego, CA). Manufacturer’s recommendations were followed, and a titer of 1:32 or greater was considered positive. Testing for arboviruses common to California was performed at the Center for Vectorborne Diseases (University of California-Davis). West Nile virus titers were determined using an indirect enzyme immunoassay (EIA) as previously described [75–78]. West Nile Virus cross reacts with St. Louis encephalitis virus (SLEV) and Western equine encephalitis virus on EIA, so EIA positive samples were tested using end-point plaque neutralization (PRNT) assays using the NY99 strain of WNV and the Kern 217 strain of SLEV. The PRNT assays were performed using >75 plaque forming units of virus grown on Vero cell culture. To be considered positive, sera had to neutralize >90% of the virus in at least a 1:4 dilution. Sera from free-flying California condors were also evaluated for active WNV infection by real time-polymerase chain reaction (rt-PCR) using primers as previously described [79, 80]. Additionally, free-flying condors that had a high WNV titer (≥ 1:256) were also screened for active WNV infection by PCR on whole blood (n = 9).

Liver and lung tissue samples collected from 14 condors that died of various causes between 1997 and 2009 were also selected for detection of a subset of potential pathogens. These 14 condors had all been in captivity and in the wild at different points in their lives. Representative cases were selected for analysis based on availability of frozen tissues at San Diego Zoo Safari Park and degree of autolysis. Tissue samples were screened for presence of avian adenovirus, coronavirus (including infectious bronchitis virus), paramyxovirus and *Mycoplasma* spp. DNA from tissues was extracted using the DNeasy Blood and Tissue kit (Qiagen, Valencia, CA, USA) following the manufacturer’s protocol. RNA was also extracted from the lung tissue samples using the QIAamp cador Pathogen Mini kit (Qiagen, Valencia, CA, USA) following the manufacturer’s guidelines. PCR primers were synthesized by Integrated DNA Technologies (San Diego, CA, USA) and were utilized in nine assays using the following primer pairs: adenovirus primers AdenokissF/AdenokissR [81], coronavirus primers Corona8pF/Corona7mR [82], IN-2F/IN-4R, Cor-p-F2/ Cor-p-R1 and Cor-p-F3/ Cor-p-R1 [83], paramyxovirus primers NCD-3/NCD-4[84], ParamyxoP1/ParamyxoPR and ParamyxoP2 /ParamyxoPR [85] and *Mycoplasma* spp. primers MycogenusF/MycogenusR [86] and MycaldP/CapaldM [87]. Positive controls were available for adenovirus and *Mycoplasma* spp.

### Statistical analysis

Seroprevalence for each pathogen was estimated along with 95% confidence intervals. The two-sided Fisher’s exact test was used to compare seroprevalences between free-flying condor release locations, between the three scavenger bird species, and between captive and free-flying condors. West Nile virus serostatus among free-flying California condors was evaluated for associations with sex, age (years), rearing site, time since last vaccination (never vaccinated, ≤ 3 years and > 3 years), number of previous vaccines (range: 0–4), and release location using logistic regression analysis (n = 85 condors). Five condors had been free-flying initially and were then brought into captivity for medical treatment for various reasons including lead exposure, microtrash, or injury. Because these individuals might differ from the others in terms of their overall health, which may affect their response to WNV vaccination, they were excluded from the analysis. Six other individuals had insufficient sample volume for WNV serology. Rearing site was defined as the location where the individual spent the majority of its first year of life: the Los Angeles Zoo; San Diego Zoo Safari Park; sites outside of California including the Oregon Zoo and the World Center for Birds of Prey in Boise, Idaho; and the wild (reference category). Each of the variables was first assessed in univariable logistic regression models. Predictor variables with p-values of ≤ 0.2 in the univariable models, in addition to potential confounding variables, were included in the multivariable analysis. Biologically plausible two-way interactions were also assessed for significance. Multivariable models were evaluated using Akaike Information Criterion (AIC), and multicollinearity among the predictor variables was assessed using variance inflation factors (VIF). Overall model fit was evaluated using the Hosmer Lemeshow test [88]. Spearman’s rank correlation was used to test the association between time since previous vaccination and age, as well as time since previous vaccination and number of vaccinations. Statistical analyses were performed using R (version 2.15.1, R-development Core team, http://www.r-project.org).

## Results

### Serosurvey

#### Free-flying condors, eagles, and turkey vultures

**Fig 2:**
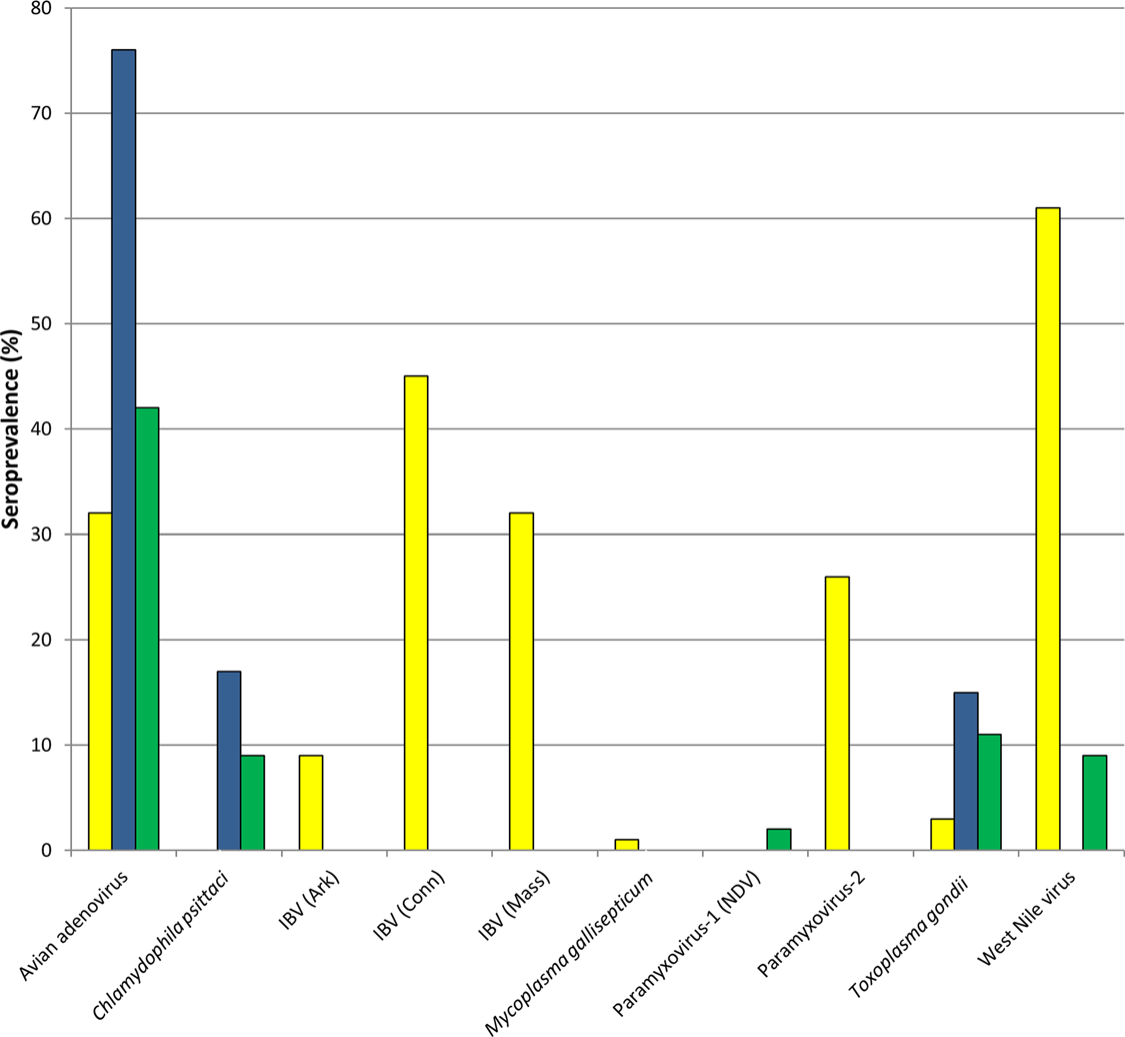
Seroprevalence of potential pathogens in free-flying California condors, golden eagles and turkey vultures in California. California Condors represented by yellow. Golden Eagles represented by blue. Turkey Vultures represented by green.

Exposure to several different potential pathogens was found in all three scavenging bird species (Fig 2). Seroprevalence varied both by location and species, depending on the particular pathogen. Overall, free-flying condors had a seroprevalence of 32% (29/92) for avian adenovirus, 9% (7/81) for IBV-Ark, 45% (38/85) for IBV-Conn, 32% (26/82) for IBV-Mass, 1% (1/92) for *M*. *gallisepticum*, 26% (22/86) for AVPM-2, and 3% (3/92) for *T*. *gondii*. The seroprevalence of WNV was 61% (55/90) in free-flying condors, and the majority of these individuals were previously vaccinated. All free-flying condors were seronegative for *C*. *psittaci*, *M*. *synoviae*, AVPM-1, AVPM-3, and avian reovirus (Table 1). Seroprevalence in golden eagles was 76% (19/25) for avian adenovirus, 17% (4/24) for *C*. *psittaci*, and 15% (4/26) for *T*. *gondii*. All eagles were seronegative for IBV, *M*. *gallisepticum*, *M*. *synoviae*, APMV-1, 2, and 3, avian reovirus, and WNV. Turkey vultures had a seroprevalence of 42% (26/62) for avian adenovirus, 9% (4/43) for *C*. *psittaci*, 2% (1/46) for AVPM-1, 11% (7/66) for *T*. *gondii*, and 9% (6/66) for WNV. All turkey vultures were seronegative for IBV, *M*. *gallisepticum*, *M*. *synoviae*, AVPM-2, AVPM-3 and avian reovirus (Table 2). Seroprevalence for each pathogen was similar among turkey vultures by site; therefore data were combined for analysis. Age and sex distributions did not differ among the three free-flying condor release locations and the vulture and eagle populations.

**Table 1:** Seroprevalence of potential pathogens in California condors from three release locations in California.

	Pinnacles National Park	Big Sur	Southern California	All release locations combined
	number positive/number tested (%; 95% CI)	number positive/number tested (%; 95% CI)	number positive/number tested (%; 95% CI)	number positive/number tested (%; 95% CI)
Avian adenovirus	4/16 (25%; 10–50%)	13/34 (38%; 29–50%)	12/42 (29%; 22–38%)	29/92 (32%; 23–42%)
*Chlamydophila psittaci*	0/16 (0%; 0–19%)	0/34 (0%; 0–10%)	0/42 (0%; 0–8%)	0/92 (0%; 0–4%)
Infectious Bronchitis Virus (Ark)	0/15 (0%; 0–20%)	3/32 (9%; 8–20%)	4/34 (12%, 10–22%)	7/81 (9%; 4–17%)
Infectious Bronchitis Virus (Conn)*	4/15 (27%; 11–52%)	9/32 (28%; 21–40%)	25/38 (66%; 52–73%)	38/85 (45%; 35–55%)
Infectious Bronchitis Virus (Mass)*	0/16 (0%; 0–19%)	8/31 (26%; 20–37%)	18/35 (51%; 40–63%)	26/82 (32%; 23–42%)
*Mycoplasma gallisepticum*	0/16 (0%; 0–19%)	1/34 (3%; 3–13%)	0/42 (0%; 0–8%)	1/92 (1%; 0.2–6%)
*Mycoplasma synoviae*	0/16 (0%; 0–19%)	0/34 (0%; 0–10%)	0/42 (0%; 0–8%)	0/92 (0%; 0–4%)
Paramyxovirus-1	0/16 (0%; 0–19%)	0/34 (0%; 0–10%)	0/42 (0%; 0–8%)	0/92 (0%; 0–4%)
Paramyxovirus-2	4/16 (25%; 10–50%)	11/31 (36%; 27–48%)	7/39 (18%; 14–27%)	22/86 (26%; 18–36%)
Paramyxovirus-3	0/16 (0%; 0–19%)	0/32 (0%; 0–11%)	0/35 (0%; 0–10%)	0/83 (0%; 0–4%)
Avian Reovirus	0/16 (0%; 0–19%)	0/34 (0%; 0–10%)	0/42 (0%; 0–8%)	0/92 (0%; 0–4%)
*Toxoplasma gondii*	1/15 (7%; 1–30%)	0/33 (0%; 0–10%)	1/42 (2%; 2–11%)	3/92 (3%; 1–9%)
West Nile virus*	8/20 (40%; 22–61%)	16/33 (49%; 37–61%)	31/37 (84%; 70–92%)	55/90 (61%; 51–71%)

CI = confidence interval

*Significant differences between release locations were found.

**Table 2:** Seroprevalence of potential pathogens in golden eagles and turkey vultures.

	Golden eagles	Turkey vultures
	number positive/number tested(%; 95% CI)	number positive/number tested(%; 95% CI)
**Avian adenovirus** *	19/25 (76%; 57–89)	26/62 (42%; 30–54)
***Chlamydophila psittaci***	4/24 (17%; 7–36)	4/43 (9%; 4–22)
**Infectious Bronchitis Virus (Ark)**	0/25 (0%; 0–13)	0/60 (0%; 0–6)
**Infectious Bronchitis Virus (Conn)**	0/25 (0%; 0–13)	0/60 (0%; 0–6)
**Infectious Bronchitis Virus (Mass)**	0/25 (0%; 0–13)	0/60 (0%: 0–6)
***Mycoplasma gallisepticum***	0/25 (0%; 0–13)	0/56 (0%; 0–6)
***Mycoplasma synoviae***	0/15 (0%; 0–20)	0/35 (0%; 0–10)
**Paramyxovirus-1**	0/25 (0%; 0–13)	1/46 (2%; 0.4–11)
**Paramyxovirus-2**	0/25 (0%; 0–13)	0/46 (0%; 0–8)
**Paramyxovirus-3**	0/25 (0%; 0–13)	0/46 (0%; 0–8)
**Avian Reovirus**	0/12 (0%; 0–24)	0/21 (0%; 0–15)
***Toxoplasma gondii***	4/26 (15%; 6–34)	7/66 (11%; 5–20)
**West Nile virus**	0/26 (0%; 0–13)	6/66 (9%; 4–18)

CI = confidence interval

* A significant difference between species was found.

Seroprevalence to IBV (Conn and Mass strains) differed among the three free-flying condor release locations. Southern California condors had a significantly higher seroprevalence of IBV-Conn (66%, 25/38) than either the Pinnacles condors (27%, 4/15, *P* = 0.015) or the Big Sur condors (28%, 9/32, *P* = 0.002). The condors released in southern California also had a higher seroprevalence of IBV-Mass (51%, 18/35), compared to those released in Pinnacles National Park (0%, 0/16, *P* < 0.001) and those released in Big Sur (26%, 8/31, *P* = 0.045). The seroprevalence to IBV-Mass was significantly higher in condors from Big Sur compared to Pinnacles National Park (*P* = 0.038).

With the exception of higher avian adenovirus seroprevalence in golden eagles compared to turkey vultures (*P* = 0.005), seroprevalence of other pathogens was not significantly different between golden eagles and turkey vultures (Table 2). Free-flying California condors had higher seroprevalence of IBV-Conn and IBV-Mass compared to turkey vultures (*P* < 0.001 for both strains) and golden eagles (*P* < 0.001 for both strains), as well as a higher seroprevalence of IBV-Ark compared to turkey vultures (*P* = 0.021). Free-flying condors also showed a significantly higher seroprevalence of APMV-2 than turkey vultures (*P* < 0.001) and golden eagles (*P* = 0.003). Conversely, a higher seroprevalence of *C*. *psittaci* was found in both golden eagles (*P* = 0.001) and turkey vultures (*P* = 0.009) relative to free-flying condors, and golden eagles had a significantly higher avian adenovirus seroprevalence than free-flying California condors (*P* < 0.001).

#### Captive condors

The condors housed at the SDZSP had a seroprevalence of 7% (2/28) for avian adenovirus, 14% (4/28) for IBV-Ark, 54% (15/28) for IBV-Mass, 57% (16/28) for *M*. *gallisepticum*, and 59% (16/27) for AVPM-2. Captive condors from SDZSP were seronegative for *C*. *psittaci*, IBV-Conn, *M*. *synoviae*, AVPM-1, AVPM-3, avian reovirus and *T*. *gondii*. Condors housed at the LAZ had a seroprevalence of 14% (2/13) for *C*. *psittaci* and were seronegative for all other pathogens. *T*. *gondii* serostatus was not determined for LAZ condors. The seroprevalence of avian adenovirus in both populations of captive condors was significantly lower than in free-flying condors (*P* = 0.018 for LAZ, *P* = 0.012 for SDZSP), as was the seroprevalence of IBV-Conn (*P* = 0.001 for LAZ, *P* < 0.001 for SDZSP), while the seroprevalence of IBV-Mass was significantly higher in SDZSP condors than in free-flying condors (*P* = 0.045). The SDZSP condors also had a higher seroprevalence of AVPM-2 and *M*. *gallisepticum* compared to free-flying condors (*P* = 0.005 and *P* < 0.001, respectively). The seroprevalence of *C*. *psittaci* was significantly higher in LAZ condors than in free-flying condors (*P* = 0.014). The seroprevalences of IBV-Mass, *M*. *gallisepticum* and AVPM-2 was significantly lower in LAZ condors compared to condors housed at SDZSP (*P* = 0.001, *P* < 0.001, *P* < 0.001, respectively).

All condor tissue samples were negative by PCR for adenovirus, coronavirus, paramyxovirus and *Mycoplasma* spp. In addition, the serum and blood samples from the free-flying condors were negative for WNV by PCR.

### Risk factors for WNV seropositivity in free-flying condors

In the multivariable regression model, original release location and rearing site were significantly associated with WNV serologic status (Table 3). Even after adjusting for age, the condors released in Southern California were over seven times as likely as those released in Big Sur to have a positive WNV titer (*P* = 0.008), and birds reared at the LAZ were over ten times as likely as birds reared in the wild to have a positive WNV titer (*P* = 0.038). The model demonstrated good fit (Hosmer Lemeshow test; *P* = 0.47). In the univariable logistic regression models, age was significantly associated with WNV serostatus in both the Big Sur and Southern California condors with the odds of seropositivity increasing with increasing age of the condor (*P* = 0.040 and = 0.007, respectively). In addition, time since vaccination was also associated with WNV serostatus in all condors, with individuals that were vaccinated greater than three years prior to sample collection more likely to be seropositive compared to condors that had never been vaccinated (*P* = 0.003). However, age and time since vaccination were not significant in the multivariable logistic regression model. An increasing number of previous vaccinations was positively associated with WNV seropositivity in the univariable models, though the association was not statistically significant (*P* = 0.194). Number of previous vaccinations was not included in the final model because it was significantly correlated with time since vaccination (ρ = 0.493, *P* < 0.001) and inclusion of the variable in the model did not improve model fit. In the bivariate analyses, age and time since vaccination were correlated (ρ = 0.392, *P* < 0.001). Age confounded the association between rearing site and WNV serologic status and was therefore included in the model.

**Table 3:** Multivariable logistic regression model evaluating the association between potential predictors and West Nile virus serostatus in free-flying California condors.

Variable	n	Odds Ratio	95% CI	Coefficient	SE	z value	p-value
**Age (in years)**	85	1.10	(0.9, 1.3)	0.09	0.10	0.96	0.338
**Release Location**							
**Big Sur**	33	ref	ref	ref	ref	ref	ref
**Pinnacles National Park**	20	1.25	(0.3, 4.9)	0.23	0.69	0.33	0.741
**Southern California**	32	7.81	(1.6, 37.3)	2.06	0.80	2.58	0.010
**Rearing Site** ^**€**^							
**Wild**	14	ref	ref	ref	ref	ref	ref
**LAZ**	23	10.23	(1.1, 96.7)	2.33	1.15	2.03	0.042
**SDZSP**	28	6.08	(0.7, 53.1)	1.80	1.11	1.63	0.103
**OUT**	20	4.31	(0.6, 28.9)	1.46	0.97	1.50	0.133
**Time since last vaccination**							
**No previous vaccination**	16	ref	ref	ref	ref	ref	ref
**1–36 months**	42	0.99	(0.2, 4.2)	-0.01	0.73	-0.01	0.993
**> 36 months**	27	3.03	(0.6, 15.8)	1.11	0.84	1.31	0.190
**AIC**		101.2					

^€^ Wild = hatched and raised in the wild, LAZ = raised at the Los Angeles Zoo, SDZSP = raised at San Diego Zoo Safari Park, OUT = raised outside of California, SE = standard error, CI = confidence interval, ref = reference category

## Discussion

This study provides preliminary baseline data on seroepidemiologic status for potential pathogens in California condors, turkey vultures and golden eagles. Though the individuals in this study were apparently healthy when sampled [14, 74], several of the pathogens we identified in these species have caused severe clinical disease in other avian species, including poultry and raptors.

Except for birds housed at the LAZ, condors had a high seroprevalence of two strains of IBV, while there was no evidence of exposure in turkey vultures and golden eagles. Because the test used for IBV is not validated in any of the species tested here, it is possible that antibodies to similar viruses were cross-reacting with the IBV tests. Infectious bronchitis virus, which is primarily a poultry pathogen, is a group 3 coronavirus and these viruses, including some genetically similar to IBV, have been detected in various wild bird species [54, 55]. Also, there is evidence that turkeys can be infected experimentally by group 2 coronaviruses [89]. At this time, it is unknown whether the virus responsible for the high seroprevalence of IBV is specific to condors or if it has spilled over from another species. Further investigation is needed to understand these serologic results and characterize coronaviruses that could be infecting condors.

Similar to IBV, both free-flying condors and condors at the SDZSP had relatively high seroprevalences of APMV-2 (26% and 59%, respectively), while turkey vultures, golden eagles and condors at the LAZ were seronegative. Commercial poultry, which is commonly fed to captive raptors, has been reported to have a seroprevalence of APMV-2 between 15 and 43% [48, 90]. Exposure to avian paramyxoviruses has been found in several species of captive raptors, and domestic poultry used as food for the raptors was suspected to be the source of infection in these instances [52, 66]. Transmission of virus from wild passerines is also a possibility.

A relatively high seroprevalence of *M*. *gallisepticum* was found in condors housed at the SDZSP, while only a single free-flying condor was found to be seropositive for *M*. *gallisepticum*, and no evidence of exposure was found in turkey vultures, golden eagles or captive condors housed at the LAZ. Some condors at the SDZSP and LAZ are housed outside where there is potential for contact with house finches. Isolation and characterization of *M*. *gallisepticum* from captive condors and house finches at the zoo are needed to better understand potential disease transmission between these species. Reasons for the differences in the seroprevalence of AVPM-2, *M*. *gallisepticum* and IBV between the two captive populations are unclear, and further investigation is needed to determine the significance of these findings.

The high seroprevalence of adenovirus found in the free-flying condors (32% overall), turkey vultures (42%), and golden eagles (76%) is consistent with findings for adenoviruses in other species, including free-flying buzzards [44]. In contrast to free-flying condors, condors at the LAZ and SDZSP showed a relatively low seroprevalence (0.0% and 7.1%, respectively), suggesting free-flying scavenging birds have natural exposure to adenoviruses in the wild. Adenoviruses have a wide range of virulence, and infection can vary from subclinical disease to significant mortality [91]. Given the potential for mortality from adenoviruses, pathogen isolation and characterization using molecular methods, and continued monitoring of this pathogen are warranted.

We found that 61% of free-flying California condors were seropositive for WNV, while only 9% of turkey vultures and none of the golden eagles tested were seropositive. In the multivariable model, release location and rearing site were associated with WNV serostatus in free-flying condors, while previous vaccination for WNV was not predictive of serologic status. Southern California birds and those raised at LAZ were the most likely to be seropositive for WNV. These findings could be attributed to the geographic distribution of WNV activity in California. In 2010, the year in which the free-flying condors were sampled, Los Angeles and Kern counties in southern California had significant WNV activity in birds, horses and humans, while the western counties of central California—San Luis Obispo, Monterey and San Benito—had no WNV activity reported for the year [92].

Several of the condors in our study were only vaccinated once against WNV, which may explain the lack of association between vaccination and serostatus in the multivariable analysis. Also, the relatively small number of condors that were not vaccinated against WNV, 16, of which six were seropositive, combined with the effect of natural exposure on titer may have resulted in our inability to detect an association between vaccination and serostatus. California condors receiving two vaccinations spaced three weeks apart have been reported to produce a strong antibody response by 60 days post vaccination [93]. However, a single WNV vaccination in other susceptible species, the western scrub jay (*Aphelocoma californica*) and island scrub jay (*Aphelocoma insularis*), has been reported to have minimal effect on titer [94, 95]. Age was significantly associated with seropositivity in condors released in Big Sur and Southern California. In addition, seropositive condors had significantly greater time since vaccination compared to seronegative condors. Natural exposure to WNV has been shown to produce strong antibody responses in other avian species such as the western scrub jay [95] and WNV titers in California condors have been long lasting, up to 416 days post-vaccination [93]. Thus, it is not surprising that older condors are more likely to be seropositive for WNV as a result of strong antibody responses to WNV upon natural exposure.

While we found strong evidence of exposure for many avian pathogens, improved testing methodologies and characterization of pathogens are needed to more completely understand the true infection status, epidemiology, and potential pathogenicity of these viruses and bacteria in scavenging birds. Because the tests used in this study were not validated for the species tested, it is possible that cross-reactivity may have occurred with similar pathogens. It is also possible that some tests failed to detect antibodies, resulting in underestimation of the seroprevalences.

This study provides insight into potential pathogens present in two obligate avian scavenger species and one facultative avian scavenger species that share habitat and exhibit similar foraging behaviors in the western US. Although additional work is needed to confirm infection and further characterize the pathogens investigated here, this study is an important first step towards providing baseline data on infectious diseases in scavenging birds. The information provided by this study may prove critical, especially for conservation efforts surrounding the endangered California condor, as this species continues to expand its range in California and encounter new threats to survival.
